# Secondary Metabolites for the Reduction of Oxidative Stress

**DOI:** 10.3390/molecules28227555

**Published:** 2023-11-12

**Authors:** Andrea Ragusa

**Affiliations:** 1 Department of Biological and Environmental Sciences and Technologies, Campus Ecotekne, University of Salento, Via Monteroni, 73100 Lecce, Italy; andrea.ragusa@unisalento.it; Tel.: +39-0832-319208; 2 CNR NANOTEC—Institute of Nanotechnology, c/o Campus Ecotekne, Via Monteroni, 73100 Lecce, Italy

Oxidative stress is a condition that occurs when there is an imbalance between the production of reactive oxygen species (ROS) and the body’s ability to neutralize them [1]. ROS are normally produced inside cells, but their amount is finely counterbalanced by antioxidant enzymes, e.g., SOD, GPx, and catalase. However, when this homeostasis is interrupted and ROS levels are too high, or antioxidant levels are too low, oxidative stress can occur.

Oxidative stress has been linked to a wide range of pathologies, including cardiovascular diseases, cancer, neurological disorders (e.g., Alzheimer’s disease, Parkinson’s disease, multiple sclerosis), pulmonary diseases (e.g., asthma, chronic obstructive pulmonary disease), inflammatory diseases (e.g., arthritis, inflammatory bowel disease), and autoimmune diseases (e.g., lupus, rheumatoid arthritis) [2,3,4,5,6]. The exact mechanisms by which oxidative stress contributes to disease are complex and vary depending on the specific pathology [7]. However, it is thought that oxidative stress can damage cells and tissues in a number of ways, including damaging DNA and proteins, disrupting cellular signaling pathways, inducing inflammation, and causing cell death.

The growing body of evidence suggesting that oxidative stress may play a role in the initiation and progression of many chronic diseases has led to increased interest in developing strategies to reduce oxidative stress and protect against its harmful effects [7]. One way to reduce oxidative stress is to follow a healthy diet rich in antioxidants, substances that can neutralize ROS and protect cells from damage [8]. For example, fruit, vegetables, marine algae, and many traditional medicinal plants are rich in secondary metabolites with antioxidant properties, among others, as are many beverages obtained from natural products [9,10,11]. Several molecules contained in these products, although at low concentration, have already been shown to exert antioxidant activity, among others, both in vitro and in vivo [12].

In this Special Issue, comprising ten original research articles and four review articles, the latest research into oxidative stress and the exploitation of secondary metabolites for its reduction are reported (see List of Contributions for details).

For example, Xia and colleagues demonstrated that *Humulus lupulus* L. extracts can protect against senior osteoporosis (SOP) by inhibiting Aβ deposition and oxidative stress (contribution 1). The extract, which is used in various industries, improved learning abilities, memory impairment, and regulated antioxidant enzymes and bone metabolism proteins in mice. It also enhanced bone mineralization and reduced Aβ deposition in the brain and femur. The results suggest that hops extract could be a potential clinical application for SOP prevention and treatment.

On the other hand, Gajurel et al. studied the antioxidant capacity of extracts enriched in prenylated stilbenoids and derivatives from peanut hairy roots (contribution 2). The extracts were produced by co-treating hairy root cultures of the cultivars Hull, Tifrunner, and Georgia Green with methyl jasmonate, cyclodextrin, hydrogen peroxide, and magnesium chloride. The results showed that the cultivar Tifrunner had higher levels of stilbenoid derivatives arachidin-1 and arachidin-6. The extracts from Tifrunner showed the highest antioxidant capacity, with an IC_50_ of 6.004 µg/mL. These findings suggest that stilbenoid-rich extracts from peanut hairy roots could be potential nutraceuticals for human health.

Resveratrol, among the most studied stilbenoids, is found in grapes, wine, nuts, and berries, has numerous biological activities, and is considered a healthcare material [13,14]. It has antimicrobial, antioxidant, antiviral, antifungal, and antiaging effects. However, it suffers from low bioavailability when administered in vivo due to its susceptibility to enzyme degradation by the body’s innate immune system. Bohara et al., after summarizing the physicochemical characteristics of resveratrol and its beneficial effects, reviewed the recent advances in nanotechnological approaches for its delivery, thus overcoming its aforementioned limit (contribution 3). Finally, they also discussed resveratrol’s potential applications as a therapeutic and disease-preventing agent, such as anticancer and antiviral agents.

Zhang at al. investigated the potential of 25 flavonoids, another class of secondary metabolites synthesized mainly by plants, to protect PC-12 cells from methylglyoxal-induced damage (contribution 4). The results, together with structure–activity relationship (SAR) analysis and molecular docking studies, showed that the flavan-3-ols, and in particular EGCG, which showed the lowest EC_50_, could reduce oxidative stress via the Nrf2/Keap1/HO-1 and Bcl-2/Bax signaling pathways. These findings suggest that flavan-3-ols could be a potential dietary supplement for protection against diabetic encephalopathy, as they can help prevent neuronal-death-related memory impairment caused by methylglyoxal-induced oxidative stress and cytotoxicity.

Rahimifard and colleagues demonstrated that gallic acid (GA), a simple plant-derived polyphenol metabolite, has anti-aging properties in rat embryonic fibroblast (REF) cells and antidiabetic effects in pancreatic islet cells (contribution 5). The study found that GA decreased β-galactosidase activity and reduced inflammatory cytokines and oxidative stress markers in REF cells. It also improved the function of β cells and reduced apoptosis by inhibiting caspase-9 activity. The findings suggest that GA at low doses regulates senescence and diabetes pathways through its antioxidative stress potential and modulation of mitochondrial complex activities. This suggests that GA could be a useful dietary supplement.

Yang et al. discovered a benzoxazole derivative able to suppress the proliferation and growth of colorectal cancer (CRC) cell lines (contribution 6). It triggers caspase 3-mediated intrinsic apoptosis of mitochondria and autophagy initiation, primarily due to increased reactive oxygen species (ROS) accumulation in CRC cells, suggesting its use as a potential therapeutic agent for CRC treatment.

The *Cannabis sativa* L. plant contains phytocannabinoids, including Δ^9^-THC and CBD [15]. In their study, Salbini et al. investigated the biological effects of cannabidibutol (CBDB) and cannabidiphorol (CBDP), comprising the same terpenophenolic core as CBD but differing only in the length of the alkyl side chain, in human breast carcinoma cells expressing CB receptors (contribution 7). The results showed that CBD treatment affects cell viability, increases reactive oxygen species production, and activates cellular pathways related to ROS signaling. The biological activity of CBD homologs is significantly increased when combined with drugs that inhibit enzymes involved in endocannabinol metabolism or induce cellular stress pathways. The exact molecular mechanisms are yet to be fully elucidated.

Milk lipids are crucial nutritional components, but their health effects, particularly for animal milks, are still a concern [16]. A study by Aresta and colleagues analyzed four types of commercial milks, including two semi-skimmed animal milks (bovine and goat) and two vegetable ones (soy and rice), and their total and free lipid fractions (contribution 8). All raw milks showed higher antioxidant ability than rice, except for soy milk, which reduced ROS in Caco-2 cells. The free lipid fraction had the highest antioxidant potential in both chemical and biological tests. Goat and soy raw milks positively regulated Caco-2 cell viability after an inflammatory stimulus, but lost this effect when their total lipid fraction was tested. Only the free lipid fraction from rice milk preserved Caco-2 viability after LPS stimulation. The lipid profile of each milk could dictate its biological effects.

Extra-virgin olive oil (EVOO) is a crucial functional food due to its bioactive compounds and numerous biological activities [17,18]. The lipid and unsaponifiable fractions of EVOO reduce oxidative stress, acting on various body components [19]. The residual by-products of the olive oil production process contain significant amounts of antioxidant molecules, making it an excellent example of the circular economy. The olive mill wastewaters, leaves, pomace, and pits discharged from EVOO production are partially recycled in nutraceutical and cosmeceutical fields due to their antioxidant effect. In their comprehensive review, Mallamaci et al. provide an overview of the biological activities of these by-products, as shown by in vitro and in vivo assays, clinical trials, and current market formulations (contribution 9).

Terrestrial and aquatic photosynthetic organisms are an incredible source of natural products with a variety of interesting properties [20]. In their review article, Pradhan and colleagues presented the main phytochemicals extracted from marine algae, such as peptides, carbohydrates, and polyphenols, and their potential beneficial effects in fighting cancer, diabetes, and inflammatory diseases (contribution 10). In addition, a research study by the same group investigated the therapeutic potential of *Enteromorpha intestinalis* extracts from methanol, ethanol, and hexane under contrasting oxidative stress conditions (contribution 11). The total phenolic and flavonoid content was quantified, with ethanol yielding the best values. The extracts from protic and more polar solvents also showed the best antioxidant potential in several radical scavenging assays, with a concentration-dependent activity. The methanolic extract showed antidiabetic capacity with IC_50_ values of 3.8 µg/mL, almost as low as those obtained with acarbose. It also showed remarkable anti-inflammatory effects, with respectable IC_50_ values comparable to commercially used drugs like acetylsalicylic acid.

Healthcare-associated infections (HAIs) are a global public health issue, with multidrug-resistant (MDR) microorganisms being the most prevalent [21]. In this regard, oxidative stress could be considered an approach to treat MDR bacteria [22]. A study by Rangel et al. examining the bactericidal effect of ozone gas on various bacteria found that high concentrations of ozone inhibited the growth of all tested strains, reducing colony count and cell viability (contribution 12). This suggests that ozone-based decontamination approaches may be the future of hospital cleaning methods, as the widespread use of ozone in ICUs can help dampen the impact of HAIs.

Islam et al. examined the impact of prolonged storage of adult retinal pigment epithelial (ARPE-19) cell sheets on their metabolism, morphology, viability, and phenotype (contribution 13). The cells were stored at different temperatures (4 °C, 16 °C, 37 °C) for three weeks. The results showed that storage at 37 °C increased lactate production and glucose consumption, while pH dropped. Morphology deteriorated at 4 °C, 37 °C, and 16 °C, while viability was best preserved at 16 °C.

Cystic fibrosis (CF) patients experience higher oxidative stress, leading to increased reactive oxygen species (ROS) and a deficit of antioxidant molecules, which contributes to chronic lung damage [23]. Despite recurrent infection–inflammation cycles in CF patients generating a highly oxidative environment, studies suggest that the airways of CF patients present an abnormal proinflammatory milieu due to elevated oxidative stress and abnormal lipid metabolism. This could be related to cystic fibrosis transmembrane conductance regulator (CFTR) deficiency, which produces a redox imbalance in epithelial cells and extracellular fluids. Moliteo et al. reviewed and explained the main mechanism by which CFTR deficiency is responsible for the proinflammatory environment in CF lung patients and its potential as a therapeutic target (contribution 14).

Taken together, all these contributions provide an overview of the impact of oxidative stress on several aspects of human health and suggest different potential therapeutic approaches to its reduction.

## References

[1] Sies H. (2015). Oxidative stress: A concept in redox biology and medicine. Redox Biol..

[2] Chen Z., Zhong C. (2014). Oxidative stress in Alzheimer’s disease. Neurosci. Bull..

[3] Kattoor A.J., Pothineni N.V.K., Palagiri D., Mehta J.L. (2017). Oxidative Stress in Atherosclerosis. Curr. Atheroscler. Rep..

[4] Senoner T., Dichtl W. (2019). Oxidative Stress in Cardiovascular Diseases: Still a Therapeutic Target?. Nutrients.

[5] Michaeloudes C., Abubakar-Waziri H., Lakhdar R., Raby K., Dixey P., Adcock I.M., Mumby S., Bhavsar P.K., Chung K.F. (2022). Molecular mechanisms of oxidative stress in asthma. Mol. Asp. Med..

[6] Smallwood M.J., Nissim A., Knight A.R., Whiteman M., Haigh R., Winyard P.G. (2018). Oxidative stress in autoimmune rheumatic diseases. Free Radic. Biol. Med..

[7] Forman H.J., Zhang H. (2021). Targeting oxidative stress in disease: Promise and limitations of antioxidant therapy. Nat. Rev. Drug Discov..

[8] Tosti V., Bertozzi B., Fontana L. (2018). Health Benefits of the Mediterranean Diet: Metabolic and Molecular Mechanisms. J. Gerontol. A Biol. Sci. Med. Sci..

[9] Ragusa A., Centonze C., Grasso M.E., Latronico M.F., Mastrangelo P.F., Sparascio F., Maffia M. (2019). HPLC Analysis of Phenols in Negroamaro and Primitivo Red Wines from Salento. Foods.

[10] Clodoveo M.L., Tarsitano E., Sabbà E., Gesualdo L., Corbo F. (2021). The Med-Index: A food product labelling system to promote adherence to the Mediterranean diet encouraging producers to make healthier and more sustainable food products. Ital. J. Food Sci..

[11] Ávila-Escalante M.L., Coop-Gamas F., Cervantes-Rodríguez M., Méndez-Iturbide D., Aranda G. (2020). The effect of diet on oxidative stress and metabolic diseases-Clinically controlled trials. J. Food Biochem..

[12] Chikara S., Nagaprashantha L.D., Singhal J., Horne D., Awasthi S., Singhal S.S. (2018). Oxidative stress and dietary phytochemicals: Role in cancer chemoprevention and treatment. Cancer Lett..

[13] Quarta A., Gaballo A., Pradhan B., Patra S., Jena M., Ragusa A. (2021). Beneficial Oxidative Stress-Related *trans*-Resveratrol Effects in the Treatment and Prevention of Breast Cancer. Appl. Sci..

[14] Galiniak S., Aebisher D., Bartusik-Aebisher D. (2019). Health benefits of resveratrol administration. Acta Biochim. Pol..

[15] Gülck T., Møller B.L. (2020). Phytocannabinoids: Origins and Biosynthesis. Trends Plant. Sci..

[16] Ballard O., Morrow A.L. (2013). Human milk composition: Nutrients and bioactive factors. Pediatr. Clin. N. Am..

[17] Ragusa A., Centonze C., Grasso M.E., Latronico M.F., Mastrangelo P.F., Fanizzi F.P., Maffia M. (2017). Composition and Statistical Analysis of Biophenols in Apulian Italian EVOOs. Foods.

[18] Romani A., Ieri F., Urciuoli S., Noce A., Marrone G., Nediani C., Bernini R. (2019). Health Effects of Phenolic Compounds Found in Extra-Virgin Olive Oil, By-Products, and Leaf of *Olea europaea* L.. Nutrients.

[19] Yubero-Serrano E.M., Lopez-Moreno J., Gomez-Delgado F., Lopez-Miranda J. (2019). Extra virgin olive oil: More than a healthy fat. Eur. J. Clin. Nutr..

[20] Sansone F., Mencherini T., Picerno P., Lauro M.R., Cerrato M., Aquino R.P. (2019). Development of Health Products from Natural Sources. Curr. Med. Chem..

[21] Facciolà A., Pellicanò G.F., Visalli G., Paolucci I.A., Venanzi Rullo E., Ceccarelli M., D’Aleo F., Di Pietro A., Squeri R., Nunnari G. (2019). The role of the hospital environment in the healthcare-associated infections: A general review of the literature. Eur. Rev. Med. Pharmacol. Sci..

[22] Dua K., Rapalli V.K., Shukla S.D., Singhvi G., Shastri M.D., Chellappan D.K., Satija S., Mehta M., Gulati M., Pinto T.J.A. (2018). Multi-drug resistant Mycobacterium tuberculosis & oxidative stress complexity: Emerging need for novel drug delivery approaches. Biomed. Pharmacother..

[23] Galli F., Battistoni A., Gambari R., Pompella A., Bragonzi A., Pilolli F., Iuliano L., Piroddi M., Dechecchi M.C., Cabrini G. (2012). Oxidative stress and antioxidant therapy in cystic fibrosis. Biochim. Biophys. Acta.

